# 

**Published:** 1903-02

**Authors:** 


[illegible]EADS VETERINARY JOURNALISM IN AMERICA.

[illegible]XIV. FEBRUARY, 1903. No. 2

PUBLISHED MONTHLY AT $3 A YEAR. SINGLE COPIES, 30 CENTS.
FOREIGN SUBSCRIPTIONS $3.50 PER YEAR.

### THE JOURNAL OF COMPARATIVE MEDICINE AND VETERINARY ARCHIVES

LIBRARY
SURGEON GENERAL'S OFFICE
9 APR 1903

EDITED AND PUBLISHED BY
W. HORACE HOSKINS, D.V.S.

WITH THE ASSISTANCE OF

LEONARD PEARSON, B.S., V.M.D.,
Dean of the Veterinary Department U. of Pa.,
State Veterinarian of Pennsylvania.

M. E. KNOWLES, D.V.S.,
State Veterinarian of Montana.

S. J. J. HARGER, V.M.D.,

Professor of Veterinary Anatomy and Zootechnics in University of Pennsylvania Veterinary Department.

E. M. RANCK, V.M.D.,
Corresponding Secretary Pennsylvania State Veterinary Medical Association.

All matters relative to Subscriptions, Advertising and Publication should be addressed to
**Dr. W. Horace Hoskins, Managing Editor, 3452 Ludlow St., Philadelphia, Pa.**

Copies may be had on order of all news-dealers at home and abroad.

AN ADVERTISING MEDIUM UNEQUALLED IN THE VETERINARY FIELD

